# Applying Telemedicine to Multidisciplinary Pediatric Inflammatory Bowel Disease Care

**DOI:** 10.3390/children8050315

**Published:** 2021-04-21

**Authors:** Hilary K. Michel, Ross M. Maltz, Brendan Boyle, Amy Donegan, Jennifer L. Dotson

**Affiliations:** 1 Division of Pediatric Gastroenterology, Hepatology, and Nutrition, Nationwide Children’s Hospital, 700 Children’s Drive, Columbus, OH 43205, USA; ross.maltz@nationwidechildrens.org (R.M.M.); brendan.boyle@nationwidechildrens.org (B.B.); amy.donegan@nationwidechildrens.org (A.D.); jennifer.dotson@nationwidechildrens.org (J.L.D.); 2 Department of Pediatrics, The Ohio State University Wexner Medical Center, Columbus, OH 43210, USA; 3 The Center for Microbial Pathogenesis, The Research Institute, Nationwide Children’s Hospital, Columbus, OH 43205, USA; 4 The Center for Innovation in Pediatric Practice, Nationwide Children’s Hospital, Columbus, OH 43205, USA

**Keywords:** inflammatory bowel disease, Crohn’s disease, ulcerative colitis, multidisciplinary care, telemedicine

## Abstract

Multidisciplinary care is essential to the delivery of comprehensive, whole-person care for children and adolescents with inflammatory bowel disease (IBD). Team members may include medical, psychosocial, and ancillary providers as well as patient and family advocates. There is significant variability in how this care is delivered from center to center, though prior to the COVID-19 pandemic, most care occurred during in-person visits. At the onset of the pandemic, medical systems world-wide were challenged to continue delivering high quality, comprehensive care, requiring many centers to turn to telemedicine technology. The aim of this manuscript is to describe the process by which we converted our multidisciplinary pediatric and adolescent IBD visits to a telemedicine model by leveraging technology, a multidisciplinary team, and quality improvement (QI) methods. Finally, we put our experience into context by summarizing the literature on telemedicine in IBD care, with a focus on pediatrics and multidisciplinary care.

## 1. Introduction

Inflammatory bowel disease (IBD), comprised of Crohn’s disease and ulcerative colitis, is a chronic, immune-mediated disease that affects 1.5 to 3 million Americans, nearly a quarter of whom are diagnosed in pediatrics or adolescence [1,2,3]. In addition to requiring the care of a pediatric gastroenterologist, they also require ongoing preventative care from a primary care provider, and frequently require consultations with other pediatric subspecialists to address comorbid conditions and complications of IBD therapies [4]. In the psychosocial sphere, patients with IBD are at increased risk for mood and body image concerns, as well as academic, social and family distress [5,6,7,8,9,10,11]. For this reason, a multidisciplinary team including nurses, dietitians, social workers, and psychology providers is essential to care for the entire patient and his or her family. At Nationwide Children’s Hospital (NCH), multidisciplinary care is delivered via multiple formats including “on demand” consultations with a dietitian, social worker, or psychologist during a routine medical visit, as well as two types of standardized multidisciplinary visits; one immediately after diagnosis called IBD Teaching Day, and another, yearly health maintenance visit called an IBD Annual Visit.

Like medical providers across the globe, our pediatric IBD team was faced with converting clinic visits, including our multidisciplinary visits, to a virtual format at the onset of the COVID-19 pandemic [12]. In this manuscript, we will summarize our care delivery approach pre-pandemic during the in-person versions of these visits, then describe our process for converting to telemedicine using quality improvement (QI) methodology. Along the way, we will share lessons learned and propose future directions for investigation and maintenance of telemedicine in pediatric IBD care moving forward.

## 2. The Nationwide Children’s Hospital Multidisciplinary Care Model

At Nationwide Children’s Hospital, we provide care for nearly 700 children and adolescents with IBD, with approximately 110 new diagnoses annually. Seventy-two percent of our patient population have Crohn’s disease (*n* = 501), 24% (*n* = 168) have ulcerative colitis, and 4% (*n* = 28) have indeterminate colitis. Patients are 47% female (*n* = 328) with a median age of 17 years (SD 3.7).

As mentioned above, within 4 to 6 weeks of diagnosis, patients and their families are invited to attend IBD Teaching Day, where they meet with one of our two IBD nurse coordinators to learn about the pathophysiology of IBD, treatment options, and follow-up timeline. Additionally, nurse coordinators provide guidance regarding when to call and how to communicate with the medical team. A variety of educational resources are provided for patients and family alike. After the nursing portion of the visit, a dietitian performs a nutritional assessment and makes recommendations regarding dietary interventions and any additional nutritional labs. Patients treated with nutritional therapy or other dietary treatments are provided personalized guidance and instructions to implement dietary interventions. Next, our pediatric social worker sees the patient and family, discusses school (504 plans) and work accommodations (Family Medical Leave Act forms), and assists with insurance and financial needs. Finally, patients and families are seen by a psychologist to screen for anxiety and depression, assess quality of life, and provide supports for topics including coping, body image, sleep, and social functioning. Psychologists then recommend personalized follow-up or referrals, as needed. After the Teaching Day, any relevant details, questions, or concerns are relayed to the child’s primary gastroenterologist for follow-up.

**Figure 1:**
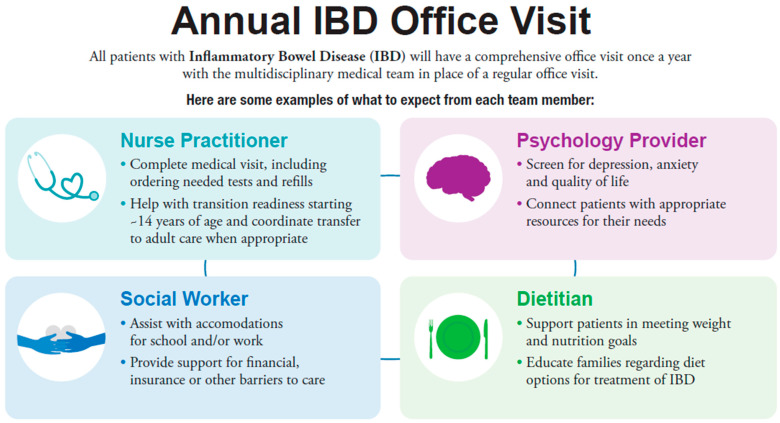
Infographic on Nationwide Children’s Hospital IBD Annual Visit Content.

Twelve months after IBD diagnosis, patients are eligible for an IBD Annual Visit. This multidisciplinary visit takes the place of a routine clinic follow-up. At this visit, our designated IBD nurse practitioner completes a routine office visit, including assessing current symptoms, completing a physical examination, and making recommendations for testing, treatment, and referrals. Individualized consultations are completed by a dietitian, psychologist, and social worker (Figure 1). Starting at age 12, our formalized transition program is delivered through the IBD Annual Visit as well.

For both multidisciplinary visit types in the in-person setting, a verbal hand-off between providers was essential. While content of the clinical visit would later be documented in the electronic medical record (EMR), a brief summary of what was covered in each individual providers’ visit, as well as any specific concerns, were shared among the team in real time in the provider work room.

## 3. Conversion from In-Person to Telemedicine

The onset of the COVID-19 pandemic in March of 2020 forced our center to convert from in-person visits to a virtual visit format. Within two weeks, IBD annual visits were conducted via telephone, with subsequent providers calling families sequentially. A barrier to this process was the perception that families often were difficult to reach via phone for subsequent visits. Approximately 4 weeks after the onset of the pandemic, some medical and psychology providers had the ability to complete video visits, but dietitians, nurses, and social workers were limited to telephone-only visits.

Within approximately 6 weeks, all providers participating in our multidisciplinary visits had access to software (Zoom video conferencing platform integrated with the EPIC EMR) and equipment (web camera, headphones, and microphones) necessary to conduct telemedicine video visits, and we were faced with the challenge of delivering efficient, high quality, comprehensive care in this new format. Barriers existed at various steps in the process, which generally fell into the following categories: scheduling, pre-visit planning, visit flow, and administration of screening tools.

In Table 1, below, we highlight each of those steps, the challenges our team faced, and the solutions we trialed. For each barrier we faced in delivering our typical multidisciplinary care, we used Plan-Do-Study-Act (PDSA) cycles, a common QI method, to (1) plan a test of change using the input of the multidisciplinary team, (2) conduct the change, (3) study the impact of the change, through qualitative feedback from team members, patients, and parents, as well as objective data such as clinic volume or rates of patients not having been seen in a particular duration of time, and (4) decide whether to adopt, adapt, or abandon the change [13]. Each solution described represents an individual intervention, though many interventions occurred concurrently. All solutions in the right-most column of Table 1 were adopted unless otherwise indicated.

**Figure 2:**
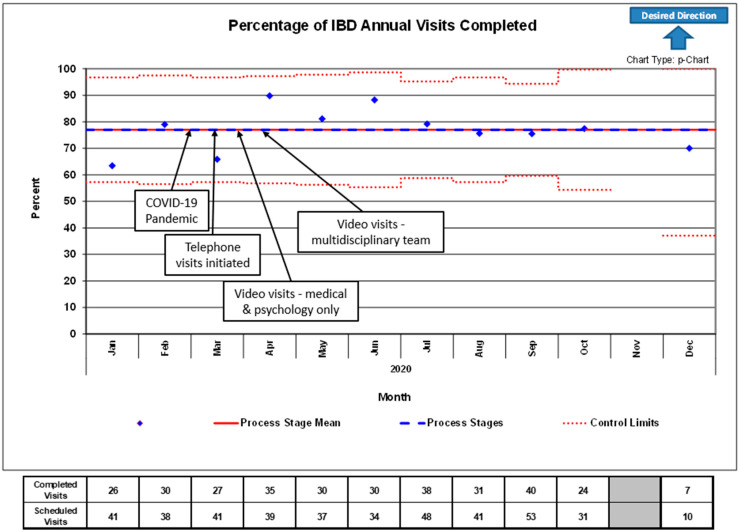
Control chart of the percentage of IBD Annual Visits completed versus scheduled in the 2020 calendar year. Arrows and text boxes indicate the process of conversion from in-person to telemedicine visits due to COVID-19. Fewer IBD Annual Visits than average were scheduled between October and December due to a team member being out for personal reasons unrelated to COVID-19 or telemedicine. Control limits represent 3 standard deviations above and below the mean.

**Figure 3:**
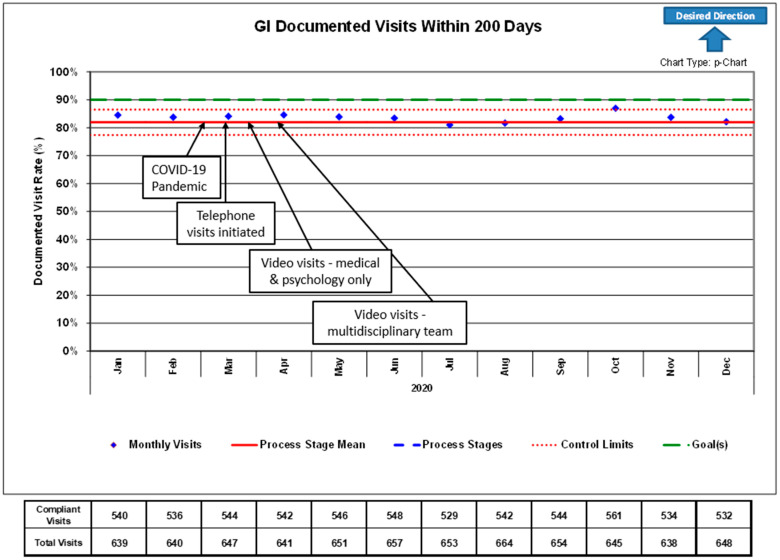
Control chart of the percentage of actively followed IBD patients with a completed gastroenterology outpatient clinic visit in the preceding 200 days during 2020. Arrows and text boxes indicate the process of conversion from in-person to telemedicine visits due to COVID-19. Control limits represent 3 standard deviations above and below the mean.

Applying these methods allowed us to use telemedicine technology to maintain Annual Visit attendance (Figure 2) and routine IBD visit attendance (Figure 3) at pre-pandemic rates. At baseline, prior to the COVID-19 pandemic, our multidisciplinary team had approximately 40 Annual Visit appointment slots per month, with a baseline completion rate between 65 and 80% due to patient no-shows. In the period of time after conversion to telemedicine, the team was able to maintain a consistent number of appointments and completion rate within pre-defined control limits. There were significantly fewer Annual Visits scheduled October through December due to a team member being out for personal reasons unrelated to COVID-19 or telemedicine.

As a quality improvement (QI) measure, our institution also measures the proportion of patients with IBD who have been seen by their provider in the preceding 200 days, with an institutional goal of 80%, which was maintained after conversion to telehealth.

## 4. Discussion

Given the chronicity and complexity IBD care, as well as the bidirectional relationship between inflammatory activity and psychosocial functioning, pediatric and adolescent patients with IBD are best served by a multidisciplinary team of medical, psychosocial, and subspecialty providers [14]. However, the onset of the COVID-19 pandemic challenged our ability to deliver multidisciplinary care in the ways we previously had, and propelled our center and others to rapidly implement telehealth options for the safety of patients, family, and providers alike [12,15].

Prior to the pandemic, the use of telemedicine in IBD care was limited and primarily in the adult setting. An aim of its application was to decrease the financial burden of IBD care; adults with IBD have annual out of pocket costs greater than two times those of adults without IBD, and costs due to missed work outweigh out of pocket costs [16]. Concerningly, medical costs associated with pediatric IBD are predicted to be higher than those with adult-onset disease [17,18,19], and data are limited regarding financial and time costs for their parents due to missed work.

Research on telehealth interventions in adult IBD care generally reveal that they are safe, feasible and acceptable by patients and providers. Additionally, they have the potential to improve disease-related knowledge, adherence, self-management skills, and quality of life [20,21,22,23,24,25] and improve access to care [26]. Telehealth has been shown to decrease frequency of office visits and decrease costs associated with travel to outpatient visits and time missed from work [22,23,25,26], without increasing flares of disease or hospitalization [23,26,27,28]. In fact, one trial showed improved disease activity and decreased hospitalizations among patients receiving telehealth and telemedicine monitoring as opposed to standard visits [27].

Several studies have sought to understand the impact of telehealth interventions in pediatric IBD care. In one study, where pediatric IBD patients were randomized to either telephone or in person follow up visits for 24 months, costs and time for consultations decreased with no inferiority in quality-of-life scores [29]. Another trial randomized patients to either usual care (clinic visits every 3 months) or a web-based remote monitoring program with one annual preplanned outpatient visit. The authors site that the eHealth program was feasible, lead to fewer days of school missed, and was not associated with increased disease activity or escalation in care [30]. One center used QI methodology to incorporate telemedicine visits and demonstrated improvement in follow-up visit frequency with favorable patient feedback [31].

Despite the many potential benefits listed above, most gastroenterology providers did not previously offer telehealth as an option due to insurance and regulatory barriers [12,15]. The existing literature seems to suggest that telehealth may be a feasible and effective tool to increase access to care, decrease burdens and costs, and potentially maintain or improve disease control in patients with IBD. As a tertiary care center seeing patients from a tri-state area, we suspect the availability of telemedicine decreased travel and time burden for many of our patients and their families. Anecdotally, our patients and their families have reported that the availability of telemedicine alleviated fears regarding the safety of travelling to the hospital for routine clinic visits during the pandemic, feelings echoed in a recent survey of pediatric patients with IBD and their parents [32]. Even now, as in person clinics have reopened, the option for telemedicine visits remains, based on provider, patient, and family preference.

The pediatric literature would benefit from trials further evaluating the impact of telemedicine on both objective and patient-reported outcomes. Specifically, there is a need for research on the incorporation and effectiveness of multidisciplinary care delivered via telemedicine in both adult and pediatric IBD care. We must be cognizant of questions of equity as we implement telemedicine as well, knowing that access to computers and internet is variable [33,34]. While the COVID-19 pandemic has brought many challenges to the way we practice medicine, the fast-tracking of telemedicine may be a change worth holding onto. Rigorous study, application of QI methodology, and incorporation of the multidisciplinary team will be essential to ensure we optimize use of this tool.

## Figures and Tables

**Table 1:** Challenges and solutions to converting multidisciplinary visits to a telemedicine format. For challenges with multiple attempted solutions, we have indicated which solutions were adopted vs. abandoned.

Step	In Person Procedures	Challenges	Telemedicine Solutions
Scheduling	All visits in person	Determination of in-person vs. telemedicine	Triaging visit types—our group developed criteria to help guide optimal visit format, acknowledging need for flexibility In-person: active or perianal disease; nutrition concerns; potential new diagnosis; no in-person visit >1 year Telemedicine: clinically stable on maintenance medication; on new medication with need to reassess symptoms; newly diagnosed with need to discuss treatment options
	Sign-up for MyChart (EMR-based patient portal) encouraged but optional	MyChart access required for telemedicine visits	When calling to schedule telemedicine appointments, administrative assistants assisted families in creating a MyChart account and provided technical support at the time of the visit
	Follow-up visits scheduled at check-out	Scheduling process	Provider sends message to administrative assistant in EMR requesting follow up interval and visit type (in-person vs. telemedicine)
	Clinic templates open 3–6 months in advance	Capacity—templates opened month-by-month	Administrative assistants keep list of patients to be scheduled with timeframe, visit type; contact families when templates open
Pre-visit planning	Chart review completed in person	Virtual processes needed	Chart review completed via secure teleconferencing
	Printed recommendations provided in clinic	Virtual processes needed	Recommendations emailed to provider ahead of patient visit
Visit flow	Sign-out between providers occurred in shared work room	Virtual sign-out process needed. Concerns regarding efficiency of visits, gaps in care, redundant care	All providers remain for entire telemedicine visit (Abandoned) Pros—Prevented redundancy, sign out not necessary, or could sign out in front of patients/caregivers Cons—Not time-efficient, took away from other clinical duties, may be unable to sign out sensitive material in front of patients/caregivers Sequential video visits with electronic sign-outs (Adopted) Pros—More time-efficient, written sign-out via EMR secure chat preferred (also trialed email but was time-consuming) Cons—Written sign-out still time consuming, concerns remain about redundancy/gaps in care delivered
	Providers could ask caregivers to step out of room for private adolescent history	Virtual process needed. Concern about patient privacy, willingness/ability of caregivers to step out	Providers simply asked parents/caregivers to step out of room and faced no challenges
Administration of screening tools	Psychosocial screening forms administered on paper prior to visit	Virtual process needed	Rights to electronic psychosocial screeners purchased, sent to families via MyChart ahead of visit, uploaded into EMR and reviewed at time of visit
Miscellaneous learnings			Telemedicine helpful to share screen with growth curves and labs Telemedicine improved accuracy of medication and dietary histories; provider could ask patient/family to get products from home for review

## Data Availability

The data presented in this study are available on request from the corresponding author. The data are not publicly available due to them being products of internal quality improvement work.
